# A revised method for measuring distraction by tactile stimulation

**DOI:** 10.12688/f1000research.4944.1

**Published:** 2014-08-12

**Authors:** Jacqueline R. Schechter, Deanna J. Greene, Jonathan M. Koller, Kevin J. Black

**Affiliations:** 1 School of Arts and Sciences, Washington University in St. Louis, St. Louis, USA; 2 Department of Psychiatry, Washington University School of Medicine, St. Louis, USA; 3 Department of Radiology, Washington University School of Medicine, St. Louis, USA; 4 Department of Neurology, Washington University School of Medicine, St. Louis, USA; 5 Department of Anatomy & Neurobiology, Washington University School of Medicine, St. Louis, USA

## Abstract

Sensory hypersensitivity (SH) refers to the tendency to attend to subtle stimuli, to persist in attending to them, and to find them noxious. SH is relatively common in several developmental disorders including Tourette Syndrome and Chronic Tic Disorder (TS/CTD). This study was an attempt to quantify the extent to which a mild tactile stimulus distracts one’s attention in TS/CTD. Fourteen adults with TS/CTD and 14 tic-free control subjects completed questionnaires regarding SH and ADHD, and TS/CTD subjects completed self-report measures of current and past tic disorder symptoms and of current obsessions and compulsions. All subjects performed a sustained attention choice reaction time task during alternating blocks in which a mildly annoying stimulus (von Frey hair) was applied to the ankle (“ON”) or was not applied (“OFF”). We present here the clinical and cognitive task data for each subject.

## Introduction

Tourette Syndrome and Chronic Tic Disorder (TS/CTD) are complex neuropsychiatric disorders in which patients present with multiple motor or vocal tics. In many patients with TS/CTD, attentional problems are present and ADHD is the most common condition that co-occurs with TS/CTD, occurring in about 50% of patients ( Greimel *et al.*, 2011). These attentional problems can range from mild to severe and can impact the patient’s ability to complete tasks, sustain attention, and keep track of personal items. Sensory hypersensitivity (SH; the tendency to be sensitive to subtle stimuli that most people would no longer attend to after habitation has occurred) is also commonly present in patients with TS/CTD ( Belluscio *et al.*, 2011). Patients with sensory hypersensitivity have difficulty tuning out otherwise neutral stimuli such as the tag in their shirt or the voices of people around them. In addition, patients with TS/CTD often experience a premonitory urge before a tic which some patients describe as a feeling of itchiness, pressure, tenseness, or energy. These tics and premonitory urges can also be distracting ( Kane, 1994).

The observation that SH can distract one from a cognitively demanding task suggested the approach we used in a small pilot study to attempt to quantify SH by examining the effects of a tactile stimulus on reaction time during a sustained attention task ( Panagopoulos *et al.*, 2013). The present study was designed to improve on some of the earlier methods and to compare subjects with TS/CTD to tic-free controls, with the goal of quantifying the extent to which sensory hypersensitivity in patients with TS/CTD affects sustained attention. To do this, participants with and without TS/CTD performed an attention task in the presence and absence of a subtle sensory stimulus.

## Methods

### Ethical approval

This study was approved by the Washington University Human Research Protection Office (IRB), proposal # 201108081.

### Participants

A convenience sample of 14 adults with TS/CTD and 14 tic-free adults participated in the study. Subjects completed a questionnaire that included age, sex, and the question, “Have you ever been diagnosed with any of the following,” with check boxes for ADHD (Attention-Deficit/Hyperactivity Disorder), learning disorder, atopic (allergic) dermatitis, OCD (Obsessive-Compulsive Disorder), Tourette syndrome, other tic disorder, and other neurological illness (to be specified by the respondent). All participants also completed the 26-item Adult Sensory Questionnaire (ASQ), developed to screen for sensory defensiveness in adults ( Kinnealey *et al.*, 1995), and the ADHD Rating Scale ( Barkley, 1998; Magnusson *et al.*, 2006). Subjects endorsing tics also rated symptom severity for the past week using the Yale Global Tic Severity Scale (YGTSS; Leckman *et al.*, 1989), the Yale-Brown Obsessive Compulsive Scale (Goodman *et al.*, 1989a,b), and the Premonitory Urge for Tics Scale (PUTS; Woods *et al.*, 2005). These data were collected using REDCap electronic data capture tools hosted at Washington University (Harris *et al.*, 2009). For tic subjects, the Diagnostic Confidence Index (DCI) was also completed; the DCI assesses typical historical features of TS ( Robertson *et al.*, 1999).

### Choice reaction time task

The subjects were seated at a fixed distance in front of a laptop computer in a darkened room. All subjects then performed a 12-minute choice reaction time task consisting of pressing one key on a computer keyboard when the capital letter ‘S’ appeared on the screen and pressing another key when the numeral 5 appeared on the screen. They were instructed to respond as quickly and accurately as possible with the right hand. The task consisted of 11 blocks; the first was a 2-minute OFF condition and the remaining blocks were each 1 minute long alternating between the ON and the OFF condition. Throughout the ON condition, a 4.74N von Frey hair was held against a point previously marked on the subject’s ankle, with ~1 Hz mild increases of pressure to just bend the von Frey hair. During the OFF condition, the von Frey hair was absent. E-Prime ^®^ 2 software was used to present stimuli and to collect all task data ( www.pstnet.com/eprime.cfm; Schneider *et al.*, 2002a,b).

A revised method for measuring distraction by tactile stimulation: Subject characteristics and task data **Subject_data2.csv:** This file includes demographic and diagnostic data for each subject. Legend: Group = self-reported diagnosis (1=TS/CTD, 2=control). ADHD hx = self-reported history of Attention-Deficit/Hyperactivity Disorder. OCD hx = self-reported history of Obsessive-Compulsive Disorder. ASQ = total score on the Adult Sensory Questionnaire. ASQ Pure = score from a subset of ASQ items (#1, 2, 4–14, 17, 23) that the authors felt were less likely to be affected by psychiatric comorbidity. See Methods for remaining abbreviations.
** task_data.7z:** This file contains a text file for each subject with output from ePrime ^®^ including accuracy and reaction time data for the attention task session for each subject. The file can be opened by 7-Zip, free and open source archiving software ( http://www.7-zip.org/).

## Data availability

*F1000Research*: Dataset 1. A revised method for measuring distraction by tactile stimulation: Subject characteristics and task data, 10.5256/f1000research.4944.d34156 ( Schechter *et al.*, 2014).
