# Supplementary material for: A revised method for measuring distraction by tactile stimulation
Source: F1000Res. 2014 Aug 12;3:188. [Version 1] doi: 10.12688/f1000research.4944.1 (PMC4176419; doi:10.12688/f1000research.4944.1)
Supplement: Peer review history

# Peer review: Referee response for version 1

**DOI:** 10.5256/f1000research.5280.r6165

**Published:** 2014-09-18

**Authors:** Euripedes Constantino Miguel, Marcelo Hoexter

**Affiliations:** 1 Department of Psychiatry, University of Sao Paulo, Sao Paulo, Brazil

This is an interesting and relevant study that examines the effects of a tactile stimulus on reaction time during a sustained attention task in patients with Tourette syndrome/chronic tic disorder (TS/CTD) vs. non-TS/CTD subjects in order to quantify the extent to which sensory hypersensitivity influences attentional outcome.

Sensory hypersensitivity has a conceptual overlap with what we call sensory phenomena ^1^^-^^4^. We define sensory phenomena by subjective uncomfortable or unpleasant sensations, feelings, and/or perceptions that may precede or accompany repetitive behaviors not only in TS but also in some common comorbid conditions such as obsessive-compulsive disorder (OCD). Types of sensory phenomena include:

1. Physical sensations (tactile and/or muscle-joint), defined as uncomfortable sensations localized in a specific region of the body (skin, muscles, or joints), which precede or occur along with repetitive behaviors;
2. “Just right” perceptions, triggered by tactile, visual, or auditory sensations, including the need for things to feel, look or sound “just right”;
3. Feelings of incompleteness (i.e., inner feeling or perception of discomfort that makes the patient do things indefinitely, or until feeling ‘complete’);
4. Energy release (i.e., generalized inner tension or energy that builds up and needs to be released by an action) and
5. Urge only (i.e., “have to do it” perception related to the repetitive behavior).

In the last 2 decades, our group has developed a brief instrument to assess the presence and the severity of sensory phenomena (the University of São Paulo Sensory Phenomena Scale) to investigate OCD phenotypic subtypes and its relationship with TS/CTD. This instrument has been validated in English ^5^. OCD patients with sensory phenomena have more comorbidity with tics, early-onset at OC symptoms, more symptoms of ordering and symmetry and low insight ^6^^,^^7^. Though the sample size of the Schechter *et al. s*tudy  is not large, it will be very interesting to examine the role of OCD (YBOCS Scores) as a confounder in attentional outcomes, as well as whether sensory hypersensitivity mediates the expression or severity of OCD symptoms. Finally, it would be interesting to observe how sensory hypersensitivity predicts TS treatment response and its comorbid conditions, as well as how specific treatments to these disorders influence sensory hypersensitivity.

Therefore, we believe that the investigation of subjective experiences such as sensory hypersensitivity is relevant to better refine the phenomenological presentation of TS/CTD patients, allowing the identification of more homogeneous subgroups and the mechanisms involved in treatment response. This understanding may help to reveal etiological factors as well as the development of more efficacious therapeutic interventions for a spectrum of patients that share common psychopathology involving sensorial experiences.

            

We are eager to read the final results of the present work.

Acknowledgement:

Both Drs Miguel and Hoexter receive a grant from the Brazilian Agencies for Research: FAPESP and CNPq.

We have read this submission. We believe that we have an appropriate level of expertise to confirm that it is of an acceptable scientific standard.

## References

[1] MiguelECCoffeyBJBaerLSavageCRRauchSLJenikeMA: Phenomenology of intentional repetitive behaviors in obsessive-compulsive disorder and Tourette's disorder.*J Clin Psychiatry*.1995;56(6) :246-2557775367

[2] MiguelECBaerLCoffeyBJRauchSLSavageCRO'SullivanRLPhillipsKMorettiCLeckmanJFJenikeMA: Phenomenological differences appearing with repetitive behaviours in obsessive-compulsive disorder and Gilles de la Tourette's syndrome. *Br J Psychiatry*.1997;170:140-145909350210.1192/bjp.170.2.140

[3] MiguelECdo Rosário-CamposMCPradoHSdo ValleRRauchSLCoffeyBJBaerLSavageCRO'SullivanRLJenikeMALeckmanJF: Sensory phenomena in obsessive-compulsive disorder and Tourette's disorder.*J Clin Psychiatry*.2000;61(2) :150-1561073266710.4088/jcp.v61n0213

[4] FerraoYAShavittRGPradoHFontenelleLFMalavazziDMde MathisMAHounieAG: Sensory phenomena associated with repetitive behaviors in obsessive-compulsive disorder: an exploratory study of 1001 patients.*Psychiatry Res*.2012;197(3) : 188253-25810.1016/j.psychres.2011.09.017Reference source22361443

[5] SampaioASMcCarthyKDMancusoEStewartSEGellerDA: Validation of the University of São Paulo's Sensory Phenomena Scale -- English version.*Compr Psychiatry*.2014;55(5) : 1881330-133610.1016/j.comppsych.2014.02.008Reference source24666717

[6] PradoHSRosárioMCLeeJHounieAGShavittRGMiguelEC: Sensory phenomena in obsessive-compulsive disorder and tic disorders: a review of the literature.*CNS Spectr*.2008;13(5) :425-4321849648010.1017/s1092852900016606

[7] ShavittRGde MathisMAOkiFFerraoYAFontenelleLFTorresARDinizJBCostaDLdo RosárioMCHoexterMQMiguelECSimpsonHB: Phenomenology of OCD: Lessons from a large multicenter study and implications for ICD-11.*J Psychiatr Res*.2014;57: 188141-14810.1016/j.jpsychires.2014.06.010Reference source25012187PMC7326117

# Response

**Published:** 2014-09-18

**Authors:** Kevin J. Black

**Affiliations:** Washington University in St. Louis, USA

We thank Drs. Miguel and Hoexter for their thoughtful, forward-looking commentary, and appreciate their suggestions on phenomenology and characterizing comorbidity. I have followed earlier reports on the USP-SPS with great interest, but was unaware of the recent publication on the English version. It would undoubtedly help place sensory hypersensitivity in better context with other sensory phenomena that occur commonly in OCD and tic disorders, and will be of interest to our future work along this line.

# Peer review: Referee response for version 1

**DOI:** 10.5256/f1000research.5280.r6060

**Published:** 2014-09-08

**Authors:** Andrea Cavanna

**Affiliations:** 1 Department of Neuropsychiatry, University of Birmingham, Birmingham, UK

This is an interesting and original research project. The methodology is sound and the quality of the data is high.

I have read this submission. I believe that I have an appropriate level of expertise to confirm that it is of an acceptable scientific standard.

# Response

**Published:** 2014-09-08

**Authors:** Kevin J. Black

**Affiliations:** Washington University in St. Louis, USA

We appreciate Prof. Cavanna's assessment.
